# Contribution of Histamine to Nociceptive Behaviors Induced by Intrathecally Administered Cholecystokinin-8

**DOI:** 10.3389/fphar.2020.590918

**Published:** 2020-10-29

**Authors:** Takafumi Hayashi, Chizuko Watanabe, Soh Katsuyama, Yasuyuki Agatsuma, Damiana Scuteri, Giacinto Bagetta, Tsukasa Sakurada, Shinobu Sakurada

**Affiliations:** ^1^ Laboratory of Pharmaceutical Sciences, Faculty of Pharmaceutical Sciences, Tohoku Medical and Pharmaceutical University, Sendai, Japan; ^2^ Department of Physiology and Anatomy, Faculty of Pharmaceutical Sciences, Tohoku Medical and Pharmaceutical University, Sendai, Japan; ^3^ Center for Clinical Pharmacology and Pharmaceutics, Nihon Pharmaceutical University, Saitama, Japan; ^4^ Preclinical and Translational Pharmacology, Department of Pharmacy, Health Science and Nutrition, University of Calabria, Cosenza, Italy; ^5^ Center for Supporting Pharmaceutical Education, Faculty of Pharmaceutical sciences, Daiichi University of Pharmacy, Fukuoka, Japan

**Keywords:** cholecystokinin-8, nociceptive behaviors, histamine, spinal cord, tachykinin neurokinin-1 receptor, *N*-methyl-D-aspartate receptor

## Abstract

The involvement of spinal release of histamine in the nociceptive behaviors induced by cholecystokinin-8 (CCK-8) was investigated in mice. Intrathecal (i.t.) injection of CCK-8 elicited the nociceptive behaviors consisting of biting and licking. The nociceptive behaviors induced by i.t. treatment with CCK-8 showed two bell-shaped patterns. The histamine H_3_ receptor antagonist significantly promoted the nociceptive behaviors induced by CCK-8 at doses of 1–100 fmol and 100 pmol. The nociceptive behaviors elicited by CCK-8 was inhibited by i.t. administration of the CCK-B receptor antagonist in a dose-dependent manner, but not by the CCK-A receptor antagonist. The nociceptive behaviors induced by CCK-8 were markedly suppressed by i.t. pretreatment with antiserum against histamine and were abolished in histidine decarboxylase-deleted gene mice. In histamine H_1_ receptor-deleted gene mice, the nociceptive behaviors induced at both 10 amol and 10 pmol of CCK-8 were not affected. The tachykinin neurokinin-1 (NK_1_) receptor antagonists inhibited CCK-8 (10 pmol)-induced nociceptive behaviors in a dose-dependent manner. CCK-8 (10 amol)-induced nociceptive behaviors was not antagonized by co-administration with the tachykinin NK_1_ receptor antagonists. The nociceptive behaviors elicited by CCK-8 were inhibited by i.t. administration of the antagonist for the *N*-methyl-D-aspartate (NMDA) receptor in a dose-dependent manner. Our results suggest that the nociceptive behaviors induced by i.t. administration of CCK-8 (10 pmol) are mediated through the spinal release of histamine and are elicited via activation of the tachykinin NK_1_ and NMDA receptors, whereas the nociceptive behaviors induced by i.t. administration of CCK-8 (10 amol) are mediated through the spinal release of histamine and elicited via NMDA receptor activation.

## Introduction

Cholecystokinin belongs to the gastrin family of peptide groups. It is widely distributed in the central nervous system and mainly present as cholecystokinin-8 (CCK-8). The sulfated octapeptide cholecystokinin, CCK-8 (H-Asp-Tyr (SO₃H)-Met-Gly-Trp-Met-Asp-Phe-NH₂) is present in the spinal dorsal horn and the primary sensory neurons in both humans and rodents (Hökfelt et al., 1985; Klein et al., 1992). Cholecystokinin A (CCK-A) receptors which are localized in the pancreatic acinar cells and cholecystokinin B (CCK-B) receptors in the stomach and brain areas, are functional membrane receptors involved in nociceptive modulation and have been identified as endogenous receptors of CCK-8 (Dufresne et al., 2006). Enhanced expression of CCK-8 mRNA in the dorsal root ganglion due to peripheral nerve injury sensitizes the primary sensory neurons, inducing nociceptive hypersensitivity (Xu et al., 1993). Moreover, CCK-8 contributes to nociceptive hypersensitivity by exciting the same neurons (Cao et al., 2012). CCK-8 exerts an antagonistic effect on opioids by decreasing morphine-induced antinociception (Noble et al., 1999), whereas morphine-induced antinociception is potentiated by pretreatment with the CCK-B receptor oligonucleotide antisense (Vanderah et al., 1994). Previous studies using a specific antagonist of the cholecystokinin receptors have shown that CCK-8 inhibited opioid antinociception through the CCK-B receptors (Dourish et al., 1990; Pu et al., 1994; Huang et al., 2007). Moreover, antagonism of the CCK-B receptor antagonists reduces burn-induced pain (Yin et al., 2016), and deletion of CCK-B receptor-deleted gene mice reduce the sensitivity of mechanical allodynia in neuropathic pain (Kurrikoff et al., 2004). Besides opioid antagonism of CCK-8, CCK-8 may have an important role as a pronociceptive peptide considering that CCK-8 has possibility to stimulate substance P-sensitive spinal neurons (Willetts et al., 1985). The above studies show that CCK-8 and cholecystokinin receptors may elicit nociceptive activation.

In the spinal cord, pain is transmitted by various nociceptive transmitters and modulators. It is well known that histamine, glutamate and substance P, and each receptor are nociceptive transmitter or modulator in the spinal cord and primary afferent neuron. Histaminergic neurons originating from the tuberomammillary nucleus in posterior hypothalamus project various brain regions, periaqueductal gray and the spinal dorsal horn, which regulate nociceptive information. (Panula et al., 1984; Panula et al., 1989; Watanabe and Yanai, 2001). These neurons are found in the superficial laminae of the spinal dorsal horn. The mRNA signals of histamine H_1_ receptor genes in the lumbar dorsal root ganglia were found in substance P and calcitonin gene-related peptidergic neurons following peripheral nerve injury in rodent (Kashiba et al., 1999). The receptors for histamine are divided into four types: H_1_, H_2_, H_3_, and H_4_ receptors. H_1_ and H_2_ receptors are postsynaptic receptors distributed in many parts of the brain (Panula and Nuutinen, 2013), whereas the location of H_3_ receptors are in the spinal cord and on the primary sensory neurons. H_3_ receptors can act as autoreceptors coupled to Gi/Go protein and control histamine synthesis and release (Morisset et al., 2000; Haas and Panula, 2003; Lin et al., 2011). H_4_ receptors are found mainly in the peripheral tissues involving in immune response and allergic reactions. Besides H_1_, H_2_, H_3_, and H_4_ receptors, histamine may modulate the response of the *N*-methyl-D-aspartate (NMDA) receptors in the central nervous system by a mechanism that does not involve H_1_, H_2_, H_3_, and H_4_ receptors (Williams, 1994; Brown et al., 2001). Histamine acting as a neurotransmitter or modulator within the brain and spinal cord, as well as on the primary neurons, can modulate pain transmission (Schwartz et al., 1991; Andrew and Craig, 2001; Sakurada et al., 2002; Hough and Rice, 2011). In behavioral research, intrathecal (i.t.) administration of histamine evoked nociceptive behaviors consisting of scratching, biting, and licking (Sakurada et al., 2003; Watanabe et al., 2008).

Glutamate receptors, both ionotropic and metabotropic, are expressed on presynaptic terminal of spinal cord, where they regulate neurotransmitter release. The spinal cord NMDA receptors play an important role in the nociceptive transmission (Dingledine et al., 1999; Sandkühler, 2000) and have attracted particular attention because of their involvement in the modulation of nociception. The primary afferent nociceptors primarily terminate in superficial laminae, whereas they connect to postsynaptic neurons in the spinal dorsal horn (Nagy et al., 2004). The NMDA receptors are heterotetrameric channels composed of seven subunits (NR1, 2A-D, and N3A + B) that form a glutamate-gated ion channel (Dingledine et al., 1999; Kew and Kemp, 2005; Ogden and Traynelis, 2011). The NR2 binding to glutamate activates a binding site to glutamate and opens the Ca^2+^/Na^+^ channel, thus resulting in neural excitation. The opening channel plays an important role in nociceptive transmission in the spinal cord (Dickenson et al., 1997). The NR1 binding to glycine are ubiquitously distributed in all laminae of the spinal cord (Nagy et al., 2004; Ogden and Traynelis, 2011), whereas NR2A, NR2B, and NR2D subunits are detected mainly in the superficial dorsal horn of the spinal cord (Boyce et al., 1999; Nagy et al., 2004). Polyamines modulate functions of the NMDA receptors through the polyamine recognition site on the NR1 subunit of the NMDA receptor complex, whereas histamine modulates polyamine recognition near site, which is called histamine binding site of the NMDA receptor (Burban et al., 2010). Histamine can imitate the action of polyamines on the NR1 subunit of the NMDA receptor ion-channel complex (Chizh et al., 2001) Glutamate released from the primary sensory neurons sensitized by noxious stimuli (Miller et al., 2011), can interact with the NMDA receptors on the cell body of postsynaptic neurons for nociceptive transmission (Verkhratsky and Kirchhoff, 2007).

It is the first report to find that i.t. administered CCK-8 induces the nociceptive behaviors, therefore the mechanism of the CCK-8-induced nociceptive behaviors is unknown.

In the current study, we found that i.t. administration of CCK-8 at extremely low doses (1 zmol–100 pmol) elicited the nociceptive behavioral response consisting of biting and licking of the hind paw and the tail along with hindlimb scratching directed toward the flank which is similar to those induced by spermine, histamine, NMDA, and tachykinin NK_1_ receptor agonists. Therefore, the mechanism of the nociceptive behaviors induced by i.t. administration of CCK-8 was investigated using antagonists of the CCK-A, CCK-B, tachykinin NK_1_, and NMDA receptors.

## Materials and Methods

### Animals

The experiments were performed with the approval of the Committee of Animal Experiments in Tohoku Medical and Pharmaceutical University and conformed to their guidelines. Male ddY mice (Japan SLC, Inc., Hamamatsu, Japan), histidine decarboxylase-deleted gene mice (Ohtsu et al., 2001), histamine H_1_ receptor-deleted gene mice (Inoue et al., 1996), and the respective wild-type mice weighing 20–24 g were used. Histidine decarboxylase-deleted gene mice and histamine H_1_ receptor-deleted gene mice were previously reported in detail (Mobarakeh et al., 2000; Yanai et al., 2003; Yoshida et al., 2005). Histidine decarboxylase-deleted gene mice and histamine H_1_ receptor-deleted gene mice were analyzed by PCR of genomic DNA from tail biopsies in order to verify whether the histidine decarboxylase or H_1_ receptor were absent in mice. The wild-type mice corresponding to respective deleted gene mice were used as controls. They were housed in cages with 5–6 weight-matched animals and placed in a colony room. Animals were housed with free access to standard food (F-2, Funabasi Farm, Co., Funabashi, Japan) and tap water in an air-conditioned room under a constant 12:12 h light/dark cycle (light on at 7:00 AM and off at 7:00 PM) at 22–24°C and 50–60% relatively humidity. Animals were used after breeding in the examination room for at least 2 days.

### Drugs and Treatment

The drugs used were CCK-8 (cholecystokinin octapeptide (sulfated) ammonium salt, Bachem, Bubendorf, Switzerland), 2-[[[4-(2-chlorophenyl)-2-thiazolyl]amino]carbonyl]-1*H*-indole-1-acetic acid (SR27897, Tocris Bioscience, Bristol, United Kingdom), 4-[[(1*R*)-2-[[(2*R*)-3-(1*H*-indol-3-yl)-2-methyl-1-oxo-2-[[(tricyclo [3.3.1.13,7]dec-2-yloxy)carbonyl]amino]propyl]amino]-1-phenylethyl]amino]-4-oxobutanoic acid (CI-988, Tocris Bioscience), histamine monoclonal (mouse) antibody (Bertin Bioreagent, Montigny le Bretonneux, France), thioperamide (Sigma-Aldrich, St. Louis, MO, United States) (2*S*,3*S*)-3-(2-methoxybenzylamino)-2-phenylpiperidine dihydrochloride (CP-99994, Sigma-Aldrich) [Tyr^6^, D-Phe^7^, D-His^9^]-substance P (6–11) (sendide, Enzo Life Sciences, Farmingdale, NY, United States), *N*-(Bz)Ala-Ala-D-Trp-Phe-D-Pro-Pro-Nle-NH_2_ (GR94800, Tocris Bioscience), agmatine sulfate (Tocris Bioscience), arcaine (Tocris Bioscience) (5*S*,10*R*)-(+)-5-methyl-10,11-dihydro-5*H*-dibenzo [*a*,*d*]cyclohepten-5,10-imine maleate (MK-801, Tocris Bioscience), D-(-)-2-amino-5-phosphonopentanoic acid (D-APV, Tocris Bioscience), and (*RS*)-3-(2-carboxypiperazin-4-yl)-propyl-1-phosphonic acid (CPP, Tocris Bioscience). All other drugs were dissolved in artificial cerebrospinal fluid (aCSF, Tocris Bioscience). Intrathecal injection (i.t.) was performed following the method described by Hylden and Wilcox (Hylden and Wilcox, 1980). The volume injected was 5 μL.

### Behavioral Procedures

To reduce of variability, mice were acclimated to individual plastic observation cages (22.0 × 15.0 × 12.5 cm) for approximately 1 h before i.t. injection. Immediately after the i.t. injection, the mice were placed in the transparent cage and the accumulated response time of biting and/or licking of the hindpaw and the tail, and along with reciprocal hindlimb scratching, was measured for 30 min at 5 min intervals using a stopwatch. The total response times of these nociceptive behaviors were pooled and recorded as single value for each animal.

### Statistical Analysis

The time of nociceptive behaviors for each group was presented as the mean ± S.E.M. Each data was calculated with a computer-associated curve-fitting program (GraphPad Prism version 8.4.3; GraphPad Software, San Diego, CA, United States). Statistical significance of the differences between the groups was established using Dunnett’s test for multiple comparisons after analysis of variance (ANOVA).

## Results

### Cholecystokinin-8-Induced Nociceptive Behaviors and Effects of Antagonists for the Cholecystokinin-A and Cholecystokinin-B Receptors on Nociceptive Behaviors Induced by Cholecystokinin-8

**FIGURE 1:**
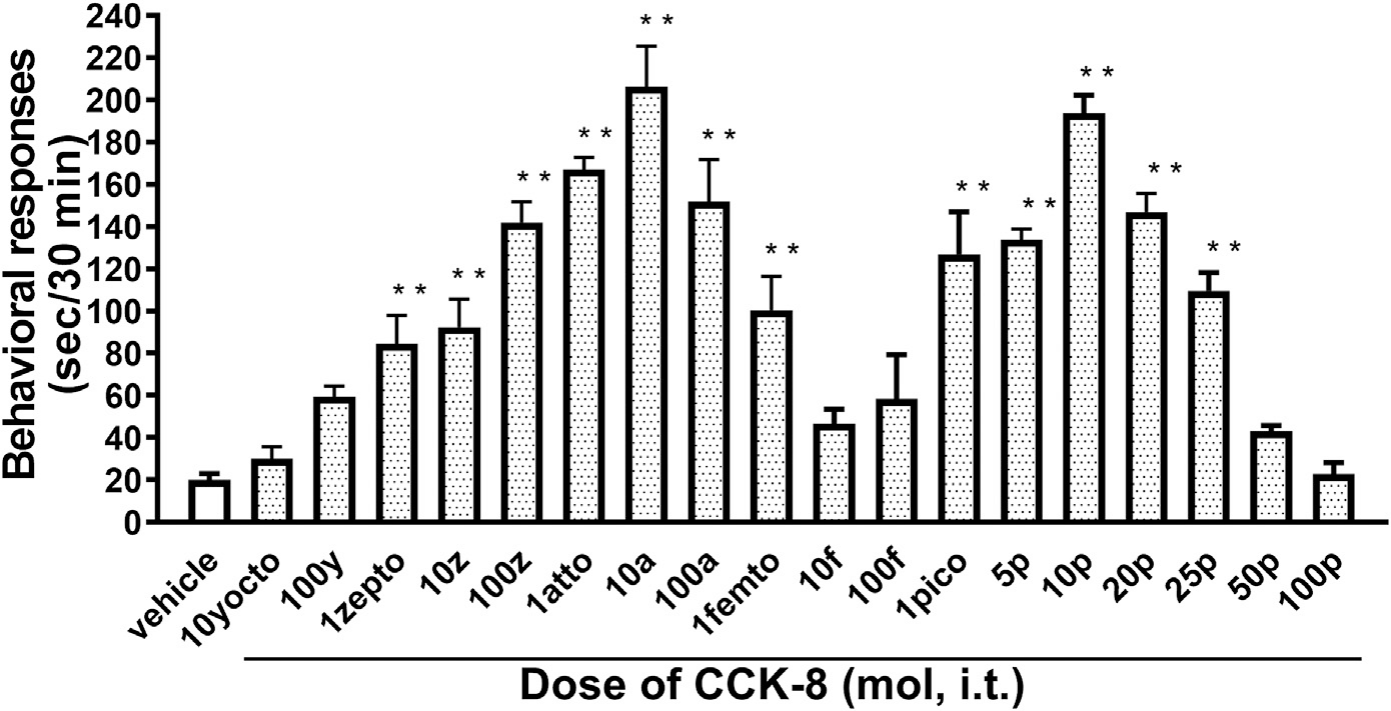
Effect of varying doses of i.t. injected CCK-8. Groups of mice were administered CCK-8 i.t., and nociceptive behaviors induced by CCK-8 were observed for 30 min. Each value represents the mean ± S.E.M. of 10 mice in each group. *F*-value of the one-way ANOVA is *F* [18, 171] = 23 (*p* < 0.0001). **p < 0.01 when compared with vehicle-control.

**FIGURE 2:**
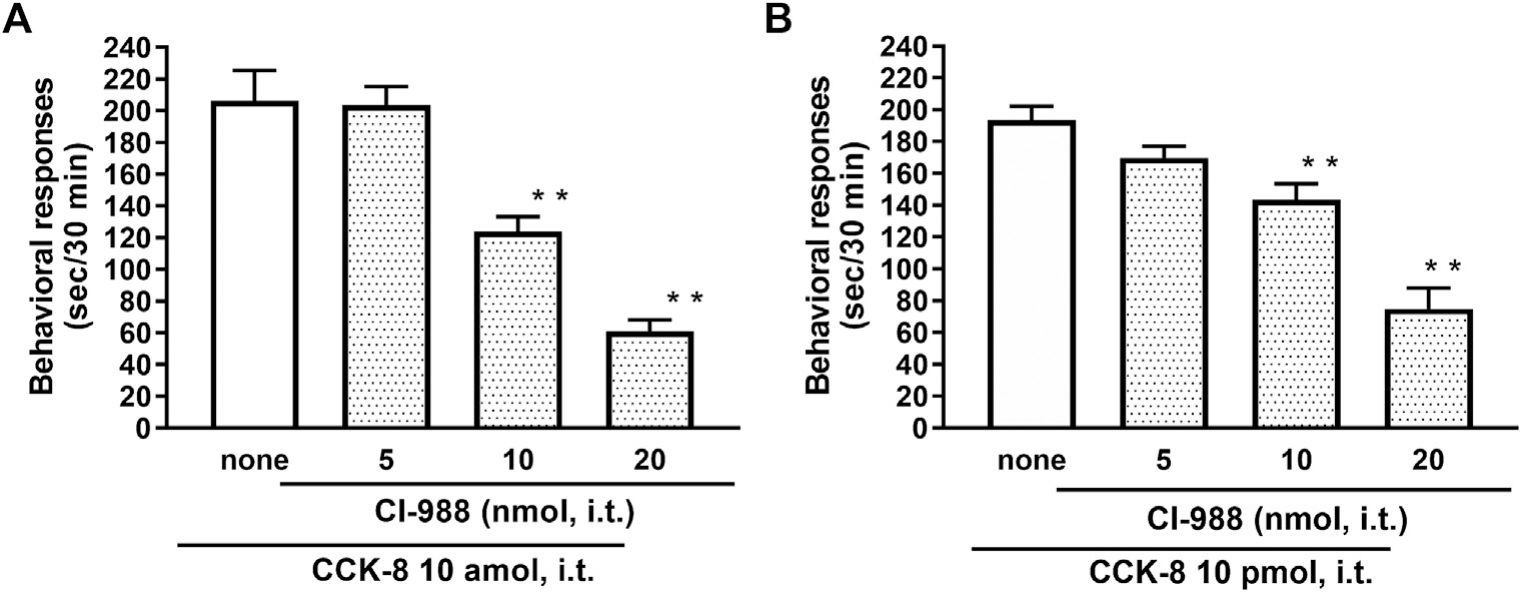
Effect of CI-988 (CCK-B receptor antagonist) on CCK-8 (**(A)**: 10 amol **(B)**: 10 pmol)-induced behavioral responses. CI-988 was co-administered i.t. with CCK-8, and nociceptive behaviors were observed for 30 min. Each value represents the mean ± S.E.M. of 10 mice in each group. *F*-values of the one-way ANOVA are **(A)**; *F* [3, 36] = 30.74 (*p* < 0.0001) **(B)**; *F* [3, 36] = 25.96 (*p* < 0.0001). **p < 0.01 when compared with CCK-8 alone.

The nociceptive behaviors induced by treatment with CCK-8 were observed in mice. I.t. injection of CCK-8 elicited the nociceptive behaviors consisting of biting and licking, and a little scratching in mice. The nociceptive behaviors induced by CCK-8 were measured for 30 min. The CCK-8-induced nociceptive behaviors was evoked significantly 5–10 min after i.t. injection and reached a maximum at 20–25 min. CCK-8-induced nociceptive behaviors showed bell-shaped patterns with two peaks from 1 zmol to 1 fmol and from 1 to 25 pmol, while the maximum effect of CCK-8 was observed at 10 amol and 10 pmol (Figure 1). The nociceptive behaviors elicited by CCK-8 (10 amol and 10 pmol) was inhibited by i.t. administration of the CCK-B receptor antagonist CI-988 in a dose-dependent manner (Figure 2). The CCK-A receptor antagonist, SR-27897, at a dose of 10 nmol had no effect on the response elicited by CCK-8 (Table 1).

**TABLE 1:** Effect of SR27897 (CCK-A receptor antagonist) on CCK-8-induced nociceptive behaviors in mice.

Treatment	Mean ± S.E.M.
CCK-8 (10 amol)	206.3 ± 19.1
+ SR-27897 (10 nmol)	178.9 ± 14.4
CCK-8 (10 pmol)	193.6 ± 8.6
+ SR-27897 (10 nmol)	175.2 ± 15.5

The nociceptive behaviors induced by i.t. injection of CCK-8 was observed for 30 min. SR27897 was co-administered i.t. with CCK-8 (10 amol or 10 pmol). The total time spent in nociceptive behaviors (s) for 30 min is represented as the mean ± S.E.M.

### Involvement of Histamine Release on Cholecystokinin-8-Induced Nociceptive Behaviors

**FIGURE 3:**
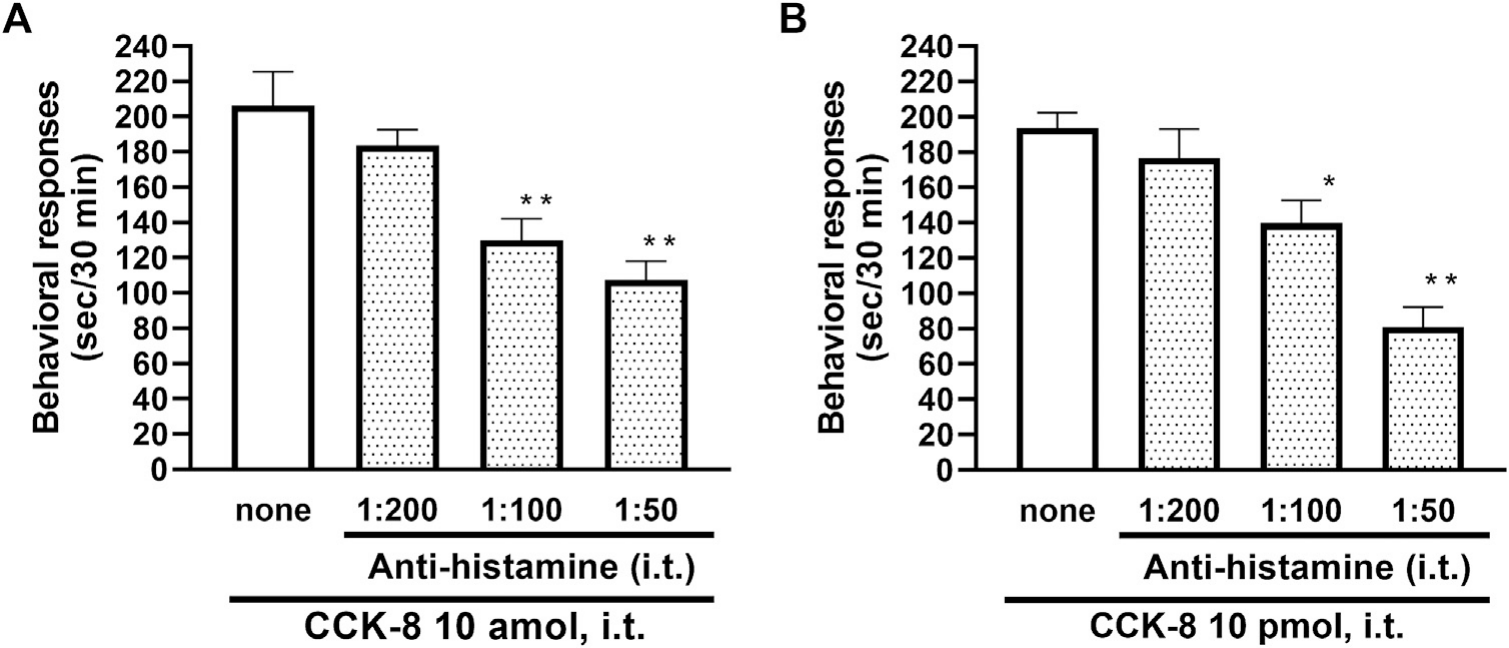
Effect of histamine antibody (anti-histamine) on CCK-8 (**(A)**: 10 amol **(B)**: 10 pmol)-induced behavioral responses. Anti-histamine was treated i.t. 5 min prior to CCK-8 treatment, and nociceptive behaviors induced by CCK-8 were observed for 30 min. Each value represents the mean ± S.E.M. of 10 mice in each group. *F*-values of the one-way ANOVA are **(A)**; *F* [3, 36] = 11.87 (*p* < 0.0001) **(B)**; *F* [3, 36] = 15.52 (*p* < 0.0001). **p < 0.01 and *p < 0.05 when compared with CCK-8 alone.

**FIGURE 4:**
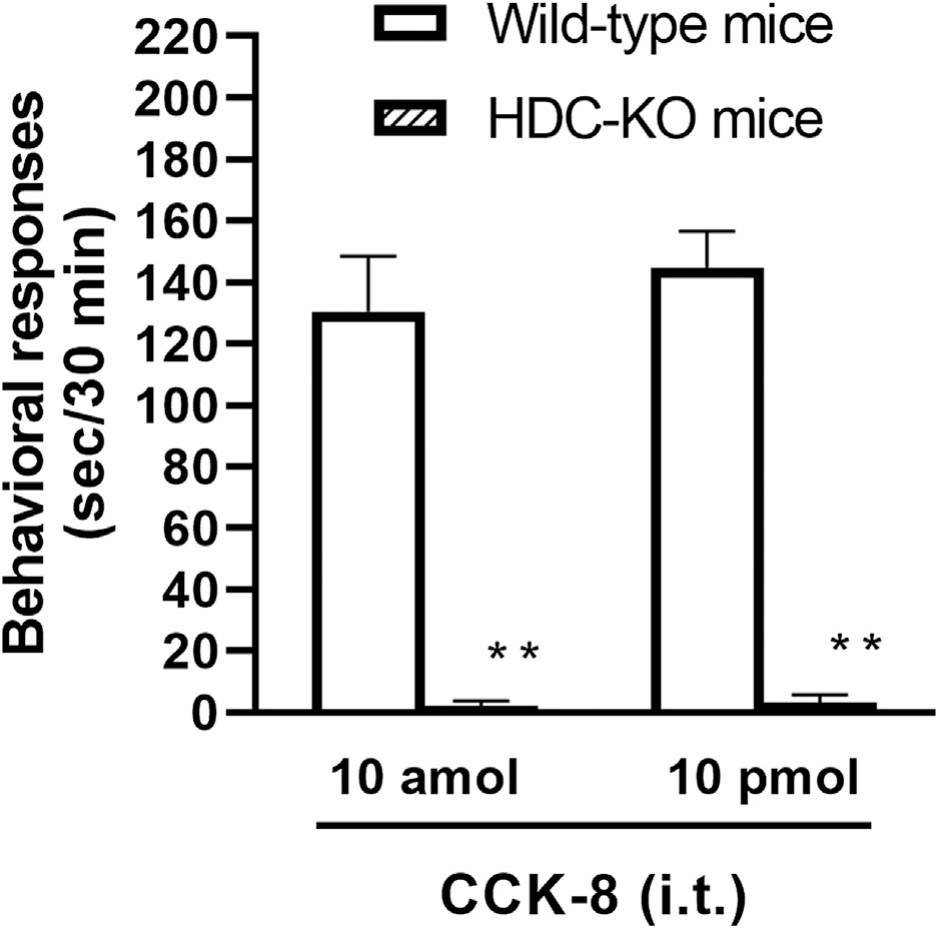
CCK-8 (10 amol, 10 pmol)-induced nociceptive behaviors in histidine decarboxylase-deleted gene mice (HDC-KO) and their wild-type mice. Groups of mice were administered CCK-8 i.t., and nociceptive behaviors induced by CCK-8 were observed for 30 min. Each value represents the mean ± S.E.M. of 10 mice in each group. **p < 0.01 when compared with wild-type mice.

To clarify the involvement of spinal release of histamine on nociceptive behaviors induced by CCK-8, the effect of an antiserum against histamine on the CCK-8-induced nociceptive behaviors was determined. Groups of mice were pretreated with i.t. antiserum against histamine (1:200–1:50 dilution) 5 min prior to i.t. treatment with CCK-8 (10 amol or 10 pmol) and the nociceptive behaviors induced by CCK-8 were measured for 30 min. I.t. pretreatment with an antiserum against histamine suppressed the CCK-8 (10 amol and 10 pmol)-induced nociceptive behaviors in a dilution-dependent manner (Figure 3). The involvement of endogenous spinal histamine in the nociceptive behaviors induced by CCK-8 was confirmed using histidine decarboxylase-deleted gene mice. The nociceptive behaviors induced at both 10 amol and 10 pmol of i.t. administered CCK-8 were abolished in histidine decarboxylase-deleted gene mice, compared to wild-type mice (Figure 4).

### Involvement of the Histamine Receptors on Cholecystokinin-8-Induced Nociceptive Behaviors

**FIGURE 5:**
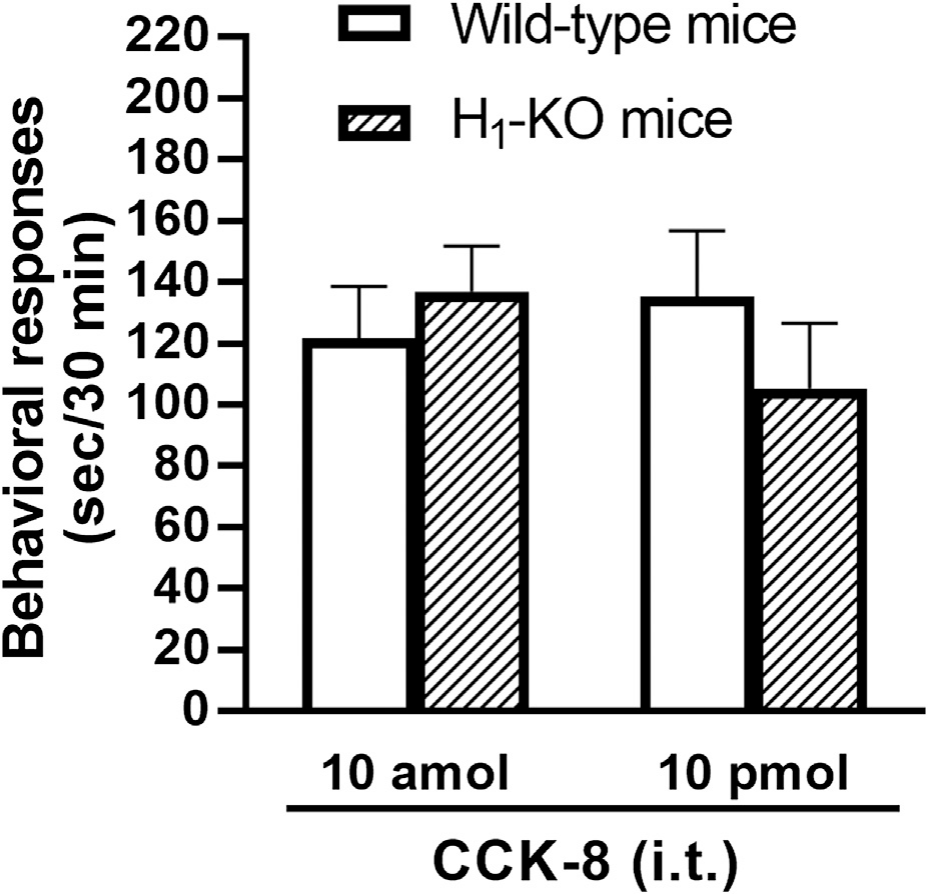
CCK-8 (10 amol, 10 pmol)-induced nociceptive behaviors in histamine H_1_ receptor-deleted gene mice (H_1_-KO) and their wild-type mice. Groups of mice were administered i.t. CCK-8, and nociceptive behaviors induced by CCK-8 were observed for 30 min. Each value represents the mean ± S.E.M. of 10 mice in each group.

The involvement of the histamine H_1_ receptors in CCK-8-induced nociceptive behaviors were determined using histamine H_1_ receptor-deleted gene mice. The nociceptive behaviors induced by i.t. administered CCK-8 at both 10 amol and 10 pmol were not affected in histamine H_1_ receptor-deleted gene mice, compared to wild-type mice (Figure 5).

**FIGURE 6:**
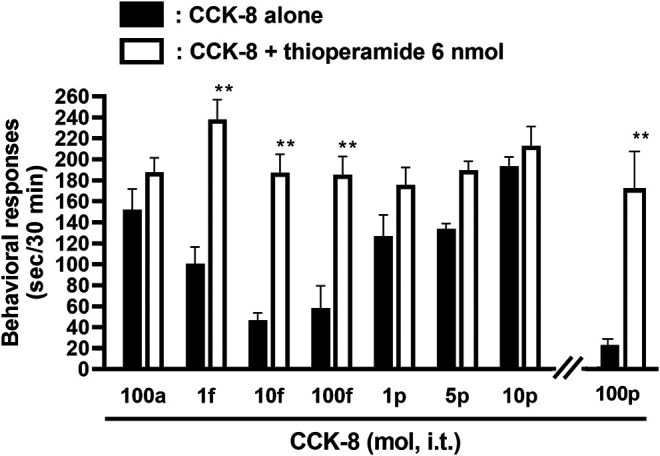
Effect of thioperamide (histamine H_3_ receptor antagonist) on CCK-8-induced behavioral responses. Thioperamide was co-administered i.t. with CCK-8, and nociceptive behaviors were observed for 30 min. Each value represents the mean ± S.E.M. of 10 mice in each group. **p < 0.01 when compared with each dose of CCK-8 alone.

The involvement of the histamine H_3_ receptors in CCK-8-induced nociceptive behaviors were determined in ddY strains of mice. Under the presence of the histamine H_3_ receptor antagonist, thioperamide at a dose of 6 nmol, CCK-8 produced similar degrees of the nociceptive behaviors at any doses (Figure 6).

### Involvement of Tachykinin Neurokinin_1_ and Neurokinin_2_ Receptor Antagonists on Cholecystokinin-8-Induced Nociceptive Behaviors

**FIGURE 7:**
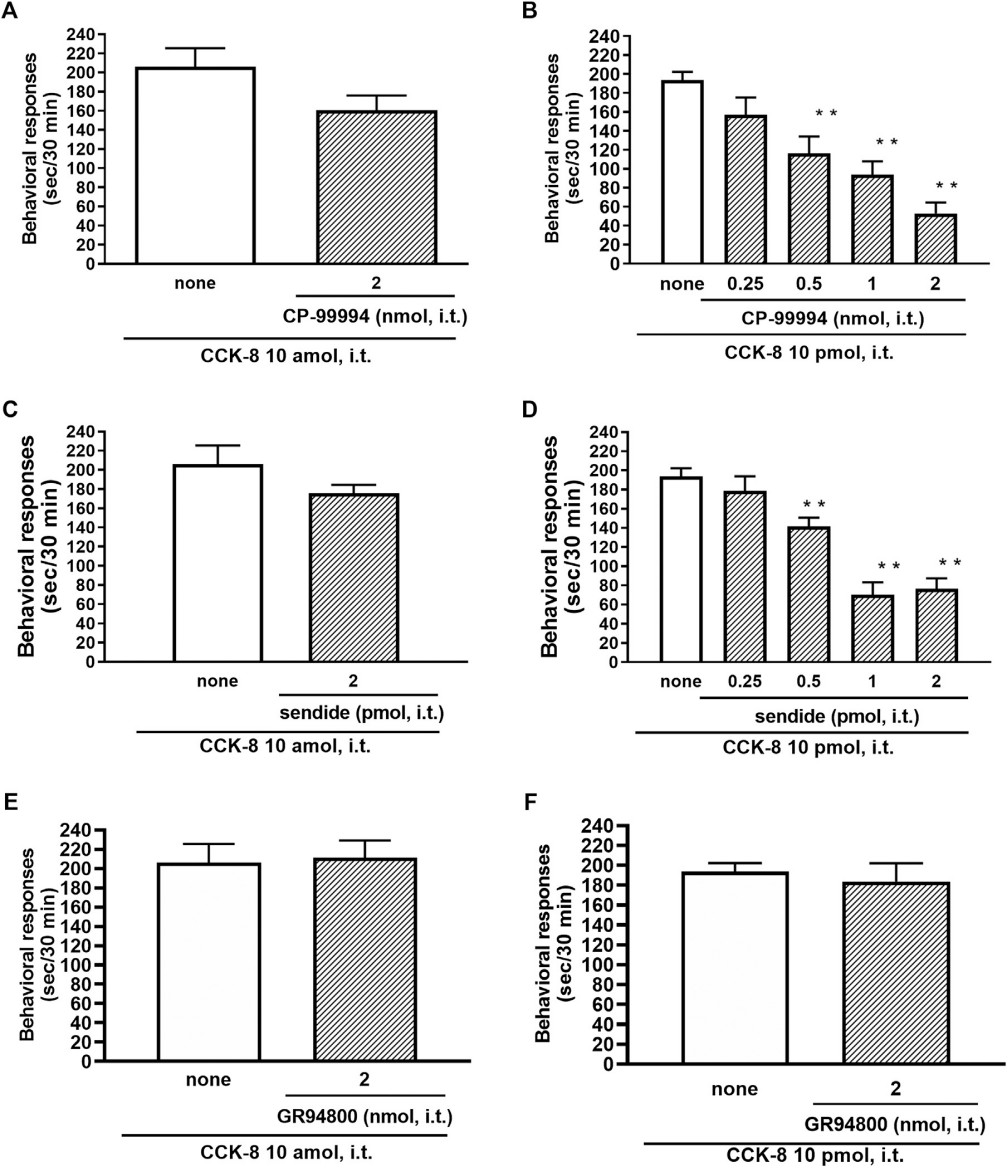
Effect of CP-99994 **(A,B)** and sendide **(C,D)** (tachykinin NK_1_ receptor antagonists), and GR94800 (tachykinin NK_2_ receptor antagonist) **(E,F)** on CCK-8 (10 amol or 10 pmol)-induced behavioral responses. CP-99994, sendide, or GR94800 was co-administered i.t. with CCK-8, and nociceptive behaviors induced by CCK-8 were observed for 30 min. Each value represents the mean ± S.E.M. of 10 mice in each group. *F*-values of the one-way ANOVA are **(B)**; *F* [4, 45] = 14.16 (*p* < 0.0001) **(D)**; *F* [4, 45] = 24.03 (*p* < 0.0001). **p < 0.01 when compared with CCK-8 alone.

The involvement of the tachykinin NK_1_ receptors on the nociceptive behaviors induced by CCK-8 were examined in mice. Groups of mice were i.t. co-administered a tachykinin NK_1_ receptor antagonist CP-99994 (0.25–2 nmol) or sendide (0.25–2 pmol) with CCK-8 (10 amol or 10 pmol). Sendide, a tachykinin NK_1_ receptor antagonist, can inhibit the substance P-induced nociceptive behaviors without affecting the nociceptive behaviors produced by the tachykinin NK_2_ and NK_3_ receptor agonists (Sakurada et al., 1992). Co-administration with CP-99994 or sendide eliminated the nociceptive behaviors induced by 10 pmol of CCK-8 (Figures 7B,D). No significant reduction of the CCK-8 (10 amol)-induced nociceptive behaviors was detected with CP-99994 or sendide (Figures 7A,C). No significant reduction of the CCK-8 (10 amol or 10 pmol)-induced nociceptive behaviors was detected on co-administration with GR94800, a selective tachykinin NK_2_ receptor antagonist (Figures 7E,F).

### Involvement of Antagonist for *N*-methyl-D-aspartate Receptor on Cholecystokinin-8-Induced Nociceptive Behaviors

**FIGURE 8:**
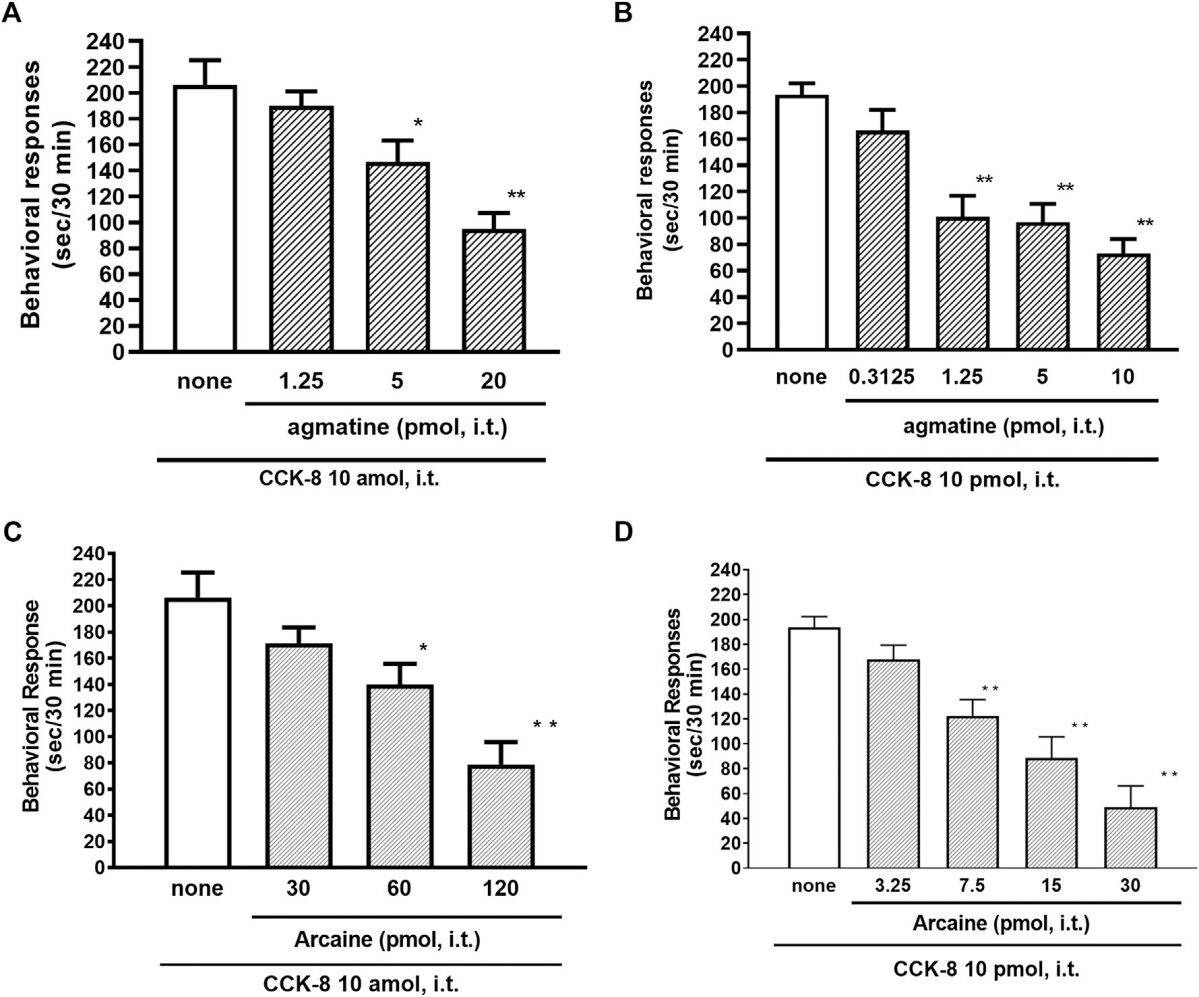
Effect of agmatine **(A,B)** and arcaine **(C,D)** on CCK-8 (10 amol or 10 pmol)-induced behavioral responses. Agmatine or arcaine was co-administered i.t. with CCK-8, and nociceptive behaviors induced by CCK-8 were observed for 30 min. Each value represents the mean ± S.E.M. of 10 mice in each group. *F*-values of the one-way ANOVA are **(A)**; *F* [3, 38] = 11.11 (*p* < 0.0001) **(B)**; *F* [4, 49] = 13.29 (*p* < 0.0001) **(C)**; *F* [3, 36] = 11.07 (*p* < 0.0001) **(D)**; *F* [4, 45] = 17.74 (*p* < 0.0001). **p < 0.01 and *p < 0.05 when compared with CCK-8 alone.

**FIGURE 9:**
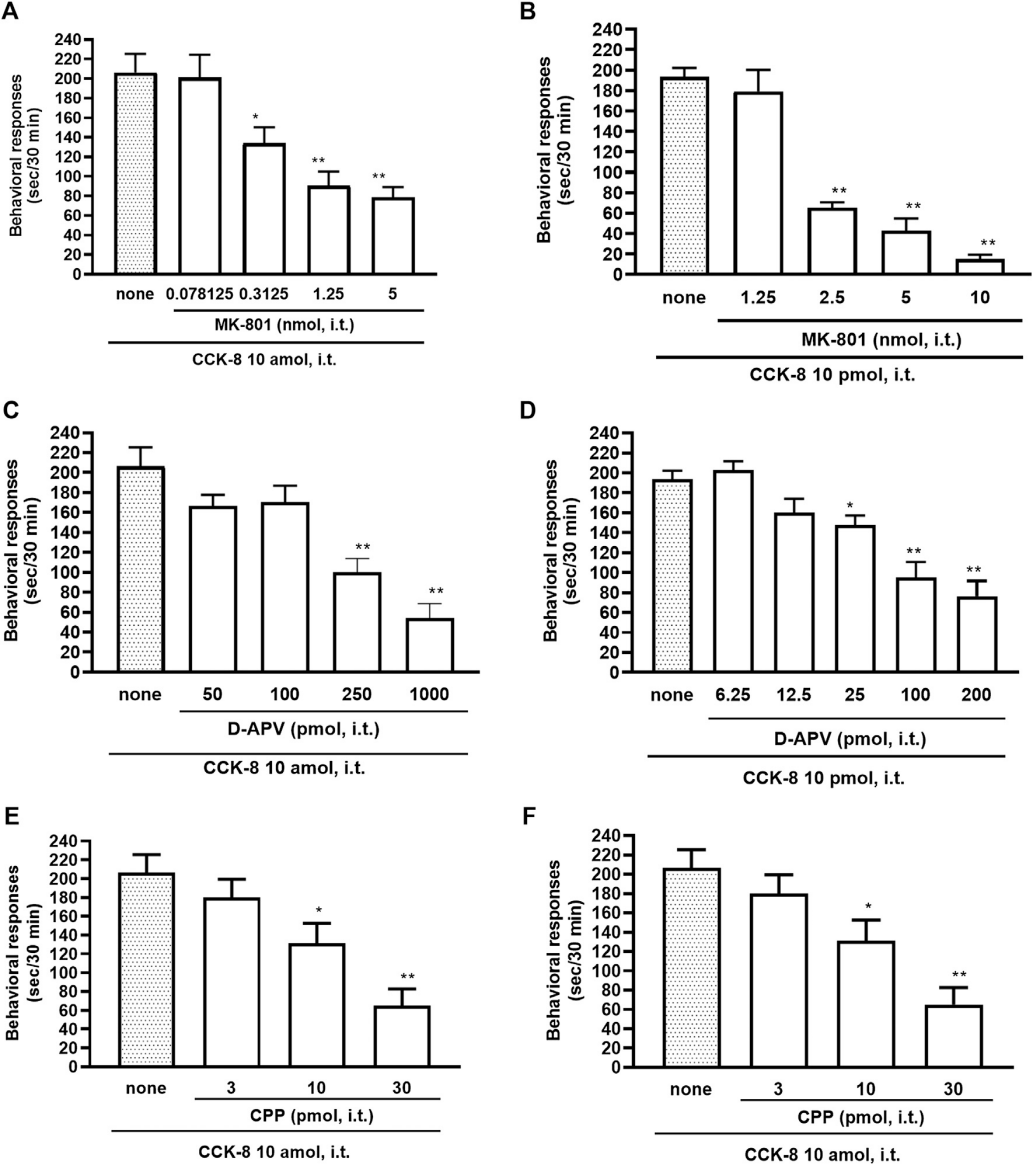
Effect of MK-801 **(A,B)**, D-APV **(C,D)**, and CPP **(E,F)** on CCK-8 (10 amol or 10 pmol)-induced behavioral responses. MK-801, D-APV, and CPP each were co-administered i.t. with CCK-8, and nociceptive behaviors induced by CCK-8 were observed for 30 min. Each value represents the mean ± S.E.M. of 10 mice in each group. *F*-values of the one-way ANOVA are **(A)**; *F* [4, 52] = 12.08 (*p* < 0.0001) **(B)**; *F* [4, 47] = 43.75 (*p* < 0.0001) **(C)**; *F* [4, 49] = 15.68 (*p* < 0.0001) **(D)**; *F* [5, 60] = 15.88 (*p* < 0.0001) **(E)**; *F* [3, 36] = 10.36 (*p* < 0.0001) **(F)**; *F* [3, 36] = 10.16 (*p* < 0.0001). **p < 0.01 and *p < 0.05 when compared with CCK-8 alone.

The NMDA receptors have multiple ligand-binding sites, having a binding site to glutamate, a binding site to glycine, an ion channel modulator-binding site, and a polyamine binding site. Therefore, the involvement of NMDA receptors on nociceptive behaviors induced by CCK-8 was examined in mice. The nociceptive behaviors induced by CCK-8 at both 10 amol and 10 pmol were attenuated by the i.t. co-administration of agmatine (0.3125–20 pmol) (Figures 8A,B) and arcaine (3.25–120 pmol) (Figures 8C,D), antagonist for NMDA receptor polyamine-binding site, in a dose-dependent manner. Co-administered MK-801 (0.078125–10 nmol) (Figures 9A,B), an antagonist for the ion channel modulator-binding site, D-APV (6.25–1,000 pmol) (Figures 9C,D) or CPP (3–30 pmol) (Figures 9E,F), antagonists for the glutamate binding site of the NMDA receptors eliminated the nociceptive behaviors induced by CCK-8 (10 amol or 10 pmol).

## Discussion

CCK-8 has been identified in the central nervous system of mammalian species. CCK-8 is distributed throughout the periaqueductal gray, ventromedial thalamus, and spinal dorsal horn, which are areas known to be associated with the nociceptive information (Baber et al., 1989). In the present study, the mechanism of the CCK-8-induced nociceptive behaviors was investigated in the mouse spinal cord. We found that i.t. administration of the CCK-8 induced nociceptive behaviors, mainly consisting of biting and licking, which are similar to that seen after i.t. injection of nociceptin or spermine (Tan-No et al., 2000; Sakurada et al., 2004; Mizoguchi et al., 2017), and i.t. administration of CCK-8 from 1 zmol to 1 fmol and from 1 pmol to 25 pmol each elicited the nociceptive behaviors in mice in two bell-shaped patterns (Figure 1). Surprisingly, 1 zmol, a minimum dose of CCK-8, significantly induced the nociceptive behaviors (Figure 1). It may be the lowest dose of any drug or compound to elicit pharmacological effect. To consider the mechanism of the CCK-8-induced nociceptive behaviors, the present study was done using 10 amol (a low dose) and 10 pmol (a high dose) of CCK-8. The nociceptive behaviors induced by i.t. administration of CCK-8 at both 10 amol and 10 pmol were antagonized by co-administered CI-988, a CCK-B receptor antagonist, and had no effect with SR27987, a CCK-A receptor antagonist (Figure 2; Table 1). Our results showed that the nociceptive behaviors induced by CCK-8 in the spinal cord were mediated by the spinal CCK-B receptor.

The involvement of the spinal release of histamine in nociceptive behaviors induced by CCK-8 was examined in mice. We found that i.t. administration of CCK-8 at both 10 amol and 10 pmol decreased nociceptive behavior after pretreatment with intrathecally administered antiserum against histamine (Figure 3). Our results showed that the nociceptive behaviors induced by i.t. administration of CCK-8 was mediated by the spinal release of histamine. This result was reinforced by the fact that the nociceptive behaviors induced by i.t. administration of CCK-8 at both 10 amol and 10 pmol was completely abolished in histidine decarboxylase-deleted gene mice (Figure 4). This finding shows that the CCK-8-induced nociceptive behaviors are caused through histamine released in the spinal cord. The nociceptive behaviors induced by intrathecally administered CCK-8 at both 10 amol and 10 pmol was not abolished in histamine H_1_ receptor-deleted gene mice (Figure 5). In the histamine receptor subtypes, H_1_, H_2_, H_3_, and H_4_ receptors, in the central nervous system, the histamine H_3_ receptor is expressed as a presynaptic autoreceptor (Schwartz et al., 1991; Arrang, 1994). Antagonism of thioperamide, a histamine H_3_ receptor antagonist, promotes the release of histamine (Itoh et al., 1991; Mochizuki et al., 1991; Barke and Hough, 1994; Jansen et al., 1998; Hough and Rice, 2011). Thioperamide significantly promoted the nociceptive behaviors induced by CCK-8 (1 fmol, 10 fmol, 100 fmol, and 100 pmol) (Figure 6). Our results show that the increase in the release of histamine induced by co-administered thioperamide could lead to enhance the CCK-8-induced nociceptive behaviors.

In this study, the nociceptive behaviors induced by i.t. administration of 10 pmol of CCK-8 were reduced by the co-administered tachykinin NK_1_ receptor antagonists CP-99994 and sendide in a dose-dependent manner (Figures 7B,D). In contrast to the nociceptive behaviors induced by i.t. administration of 10 pmol of CCK-8, the nociceptive behaviors induced by i.t. administration of 10 amol of CCK-8 were not affected by the co-administered CP-99994 at a dose of 2 nmol or sendide at a dose of 2 pmol of CCK-8 (Figures 7A,C). The histamine-induced nociceptive behaviors were inhibited by the tachykinin NK_1_ receptor antagonists (Sakurada et al., 2003). These results suggested that the nociceptive behaviors associated with i.t. administration of 10 pmol of CCK-8 were due to the release of substance P, which bind to the tachykinin NK_1_ receptors on the dorsal spinal cord. The nociceptive behaviors associated with i.t. administration of nociceptin or spermine are due to the spinal release of histamine, which are mediated by the activation of the histamine H_1_ receptors located on the primary afferent nerves and lead to the release of substance P which bind to the tachykinin NK_1_ receptors on the dorsal spinal cord (Sakurada et al., 2004; Mizoguchi et al., 2017). However, the above results demonstrated that the nociceptive behaviors induced by i.t. administration of CCK-8 at both 10 amol and 10 pmol did not involve the histamine H_1_ receptors (Figure 5).

The pharmacological similarity between substance P and CCK-8 is known on the nociceptive behaviors. CCK-8 and substance P, which are present in the capsaicin-sensitive neurons in the spinal cord, co-exist within the same fibers in the spinal dorsal horn (Dalsgaard et al., 1982; Zouaoui et al., 1990). Moreover, Willetts et al. demonstrated that spinal dorsal neurons sensitive to substance P can be excited by CCK-8 conceivably representing a pivotal nociceptive peptide in the primary sensory neuron (Willetts et al., 1985). CCK-8 was released from the spinal cord by the nociceptive stimulation (Yaksh et al., 1982). Taken together with the above reports, our current findings indicate the neuronal correlation between CCK-8 and substance P in the dorsal horn of the spinal cord. In guinea pig ileal longitudinal muscle, which mediate contractions via neurons, the CCK-8-induced contractions were mediated by the release of substance P, since a substance P antagonist reduced the maximal contraction induced by CCK-8 (Lucaites et al., 1991). Although there is no direct evidence to demonstrate that CCK-8 releases substance P, our results present the possibility that the nociceptive behaviors induced by i.t. administration of CCK-8 (10 pmol) is mediated by substance P neurons in the dorsal horn of the spinal cord, since tachykinin NK_1_ receptor antagonists reduced the CCK-8-induced nociceptive behaviors.

The NMDA receptor is a heterotetrameric protein complex consisting of seven NMDA receptor subunits (NR1, NR2A-D, and NR3A + B) that form a glutamate-gated ion channel, Ca^2+^/Na^+^ channel (Dingledine et al., 1999; Kew and Kemp, 2005; Ogden and Traynelis, 2011). The NMDA receptors have multiple ligand-binding sites, having a binding site to glutamate, a binding site to glycine, an ion channel modulator-binding site, and a polyamine-binding site. Therefore, the involvement of the NMDA receptors in the CCK-8-induced nociceptive behaviors was determined by blockers of multiple ligand-binding sites of NMDA receptor. In the present study, the nociceptive behaviors induced by i.t. administration of CCK-8 at both 10 amol and 10 pmol were dose-dependently reduced by the co-administration of antagonists for the polyamine binding site, agmatine or arcaine (Figures 8A,B), by the co-administration of an antagonist for ion channel modulator-binding site of the NMDA receptors, MK-801 (Figures 9A,B), by the co-administration of antagonists for binding site to glutamate, D-APV (Figures 9C,D) or CPP (Figures 9E,F). These results clearly show that the nociceptive behaviors associated with i.t. administration of CCK-8 at both 10 amol and 10 pmol are due to the spinal release of histamine, which leads to the release of glutamate then binding to the NMDA receptors on the dorsal horn of the spinal cord. The nociceptive behaviors induced by CCK-8 at both 10 amol and 10 pmol were strongly suppressed by agmatine and arcaine, which are antagonists for the NMDA receptor polyamine-binding site (Figure 8). I.t. high-dose histamine elicits the nociceptive behavior through the polyamine-binding site, ion channel modulator-binding site and binding site to glutamate on the NMDA receptors, but not H_1_ and H_2_ receptors, or tachykinin NK_1_ receptors (Watanabe et al., 2008). The current findings indicate that pain transmission in the spinal cord involves a complicated mutual regulation between CCK-8 and histamine. The nociceptive behaviors associated with i.t. administration of CCK-8 at both 10 amol and 10 pmol may be due to the spinal release of histamine, which preferentially and directly stimulates the polyamine-binding site of the NMDA receptors, but not the histamine H_1_ receptors, on the dorsal spinal cord.

**FIGURE 10:**
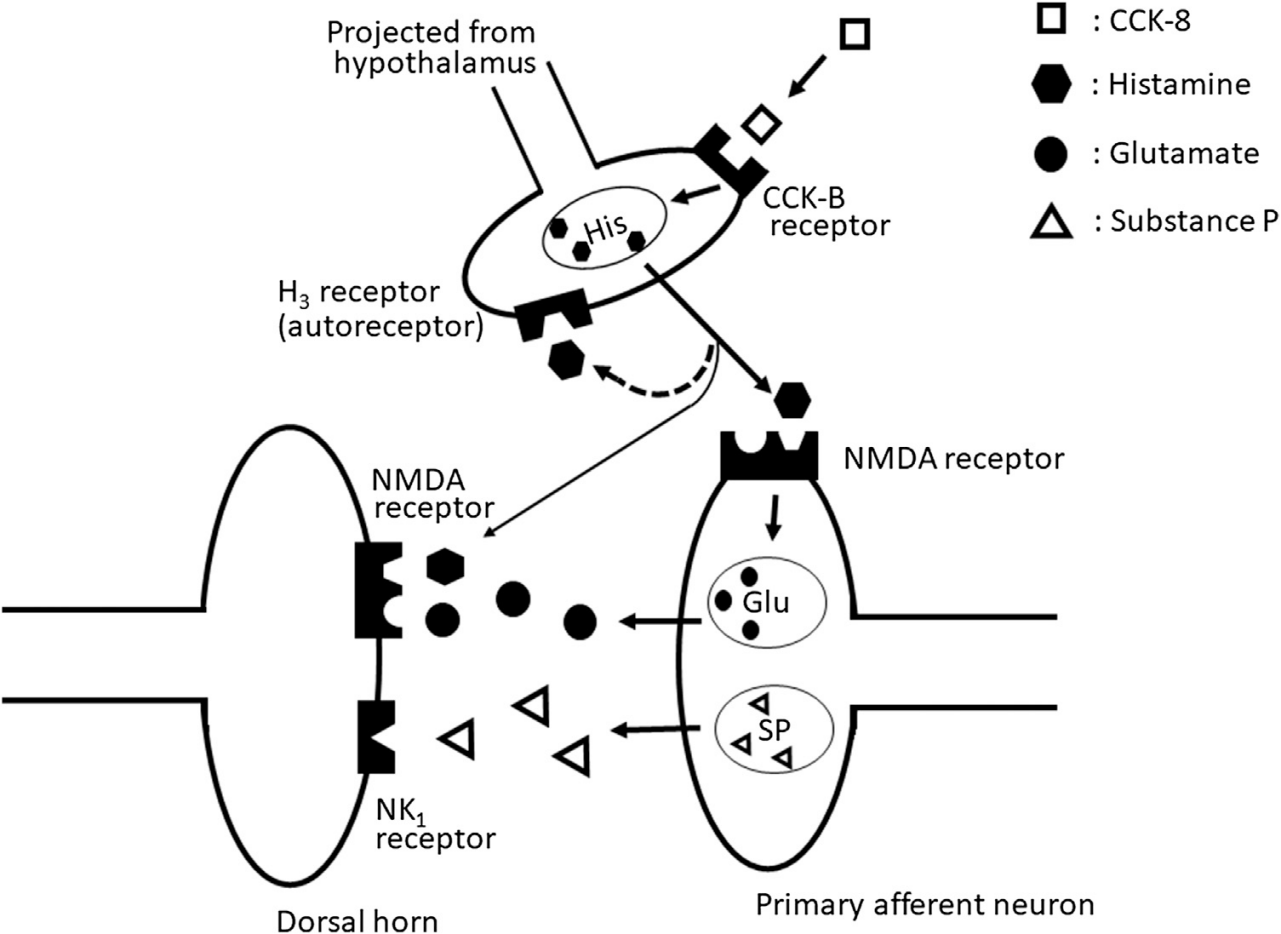
The schematic circuit diagram of the spinal dorsal horn with speculative location of the NMDA, tachykinin NK_1_, histamine H_3_, and CCK-B receptors, and their transmitter containing neuron.

Our results suggested that the nociceptive behaviors induced by i.t. administration of CCK-8 (10 pmol) are mediated through the spinal release of histamine and elicited by activating the tachykinin NK_1_ and NMDA receptors, whereas it is suggested that nociceptive behaviors induced by i.t. administration of CCK-8 (10 amol) are mediated through the spinal release of histamine and are elicited by activating the NMDA receptors (Figure 10).

## Conclusion

In conclusion, the nociceptive behaviors induced by i. t. administration of CCK-8 are mediated by the spinal release of histamine as the deletion of histidine decarboxylase gene eliminates symptom of nociceptive behavior induced by CCK-8. The released histamine directly stimulates the polyamine-binding site of the NMDA receptors as histamine acts on the polyamine-binding site of the NMDA receptors. Therefore, histamine indirectly activates the tachykinin NK_1_ and NMDA receptors via the spinal release of substance P and glutamate, respectively.

## Data Availability Statement

The raw data supporting the conclusions of this article will be made available by the authors, without undue reservation.

## Author Contributions

TH, TS, and SS were involved in protocol and project development, collected, and analyzed the data, and wrote the manuscript. CW and SK collected and analyzed the data. YA, DS, and GB were involved in protocol and project development.

## Conflict of Interest

The authors declare that the research was conducted in the absence of any commercial or financial relationships that could be construed as a potential conflict of interest.
